# Simultaneous integrated boost vs sequential boost chemoradiotherapy in anal squamous cell carcinoma: Association with improved disease-free survival and the role of treatment delivery parameters.

**DOI:** 10.1016/j.ctro.2026.101234

**Published:** 2026-07-07

**Authors:** Cécile Giannetta, Rafik Nebbache, Isabelle Etienney, Gael Goujon, Thomas Aparicio, Léon Maggiori, Laurent Abramowitz, Laurent Quéro

**Affiliations:** a Radiation-oncology department, Saint Louis Hospital, Paris, France; b Proctology department, Croix Saint Simon Hospital, Paris, France; c Gastro-enterology, Bichat Hospital, Paris, France; d Gastro-enterology Department, Saint Louis hospital, Paris, France; e INSERM U1342, Université Paris Cité, Paris, France; f Digestive surgery Department, Saint Louis hospital, Paris, France; g Proctology department, Bichat Hospital, Paris, France

**Keywords:** Anal canal cancer, radiotherapy, simultaneous integrated boost, IMRT

## Abstract

Background and purpose.

The optimal radiotherapy delivery strategy for anal canal cancer remains debated. We compared outcomes between simultaneous integrated boost (SIB) and sequential boost approaches in patients treated with chemoradiotherapy.

**Materials and methods:**

This single-centre retrospective study included consecutive patients with localised or locally advanced anal canal squamous cell carcinoma treated with volumetric modulated arc therapy between June 2019 and July 2024. All patients received 60 Gy to the primary tumour and involved lymph nodes. Prophylactic nodal irradiation was delivered either sequentially (44 Gy in 22 fractions) or with SIB (45 Gy in 30 fractions). The primary endpoint was disease-free survival (DFS). Secondary endpoints included overall survival (OS) and treatment-related adverse events (CTCAE v5.0). Survival was estimated using the Kaplan-Meier method and compared with the log-rank test. Cox models were used for multivariable analysis.

**Results:**

Eighty-four patients were included: 50 treated with SIB and 34 with a sequential approach.

Median follow-up from the end of radiotherapy was 21 and 48 months in the SIB and sequential groups, respectively. The 24-month DFS rate was 96% in the SIB group versus 70% in the sequential group. SIB was independently associated with improved DFS (HR 0.12, 95% CI 0.03–0.53; *p* = 0.006). OS at 24 months was 98% with SIB and 85% with sequential treatment, without statistical significance after adjustment. Acute grade ≥ 2 adverse events tended to be lower with SIB, without significant differences. No grade > 3 adverse event was observed. Post-treatment lymphocyte counts were higher in the SIB group (*p* = 0.029).

**Conclusion:**

SIB-based radiotherapy was associated with improved DFS without increasing the rate of treatment-related adverse events. This benefit may be partly related to shorter overall treatment time and potential immune-sparing effects. Longer follow-up and prospective studies are warranted.

## Highlights

• Simultaneous integrated boost was associated with improved disease-free survival in anal squamous cell carcinoma.
• SIB was not associated with increased acute or late treatment-related adverse events.
• The benefit associated with SIB cannot be explained solely by treatment duration.

## 1 Introduction

Anal canal cancer is a rare disease, accounting for approximately 2.5% of digestive cancers, with nearly 9900 new cases diagnosed each year in Europe [1], [2]. Over the past decades, its incidence has been steadily increasing in Western countries, particularly among women and older individuals [2], [3]. Squamous cell carcinoma is the predominant histological subtype, accounting for more than 90% of cases [4]. Persistent infection with the human papillomavirus (HPV), particularly genotype HPV16, is now recognised as the main etiological factor [5].

Concurrent chemoradiotherapy is the standard treatment for localised and locally advanced anal canal cancer, enabling excellent locoregional control and survival rates [6]. Prophylactic irradiation of regional lymph node areas is an integral part of this strategy, given the risk of microscopic dissemination [7].

Technical advances, particularly the introduction of intensity-modulated radiotherapy (IMRT), have reduced the frequency and severity of acute and late treatment-related adverse events without compromising oncological outcomes. In this landscape, the landmark phase II trial NRG Oncology/RTOG 0529 provided the main prospective support for the dose-painted IMRT approach, with significantly lower rates of acute grade ≥ 3 gastrointestinal and genitourinary treatment-related adverse events than those reported in historical 3D conformal radiotherapy series [8], [9]. By using a simultaneous integrated boost (SIB), this technique optimises dose delivery and shortens overall treatment time (OTT), potentially mitigating tumour repopulation [10].

Nevertheless, treatment-related adverse events remain frequent and may result in long-term impairment of quality of life [11], [12], [13]. This has led to increasing interest in treatment optimisation, particularly through improved treatment delivery and dose de-escalation strategies. The British PLATO platform investigates stage-adapted radiotherapy, including treatment de-escalation for lower-risk disease (ACT3 and ACT4) and dose escalation for higher-risk disease (ACT5). Recently, the phase II ACT4 trial reported that reduced-dose chemoradiotherapy achieved high 6-month complete clinical response rates with improved tolerability in patients with early-stage anal cancer, while long-term oncological outcomes remain awaited [14]. However, despite the widespread adoption of IMRT, direct comparative data between sequential boost and SIB strategies remain limited. Questions persist regarding the optimal balance between disease control, late treatment-related adverse events, and the impact of treatment delivery parameters such as OTT.

In this context, we conducted a retrospective study to compare disease-free survival (DFS) in patients with localised or locally advanced anal canal cancer treated with (chemo)radiotherapy using either a sequential boost or a SIB approach.

## 2 Patients and methods

### 2.1 Study design and population

This single-centre retrospective study included all consecutive patients with anal canal cancer treated at the radiation oncology department of Saint-Louis Hospital, Paris, France (AP-HP. Nord), between June 2019 and July 2024. Inclusion criteria were histologically confirmed squamous cell carcinoma of the anal canal, clinically classified according to the TNM Classification of Malignant Tumours, 8th Edition, as localised (T1-T2, N0) or locally advanced disease (T3-T4 or N+) and treatment with volumetric modulated arc therapy (VMAT), with or without concurrent fluoropyrimidine- and mitomycin C-based chemotherapy. Patients were treated consecutively with a sequential boost strategy until December 2021 and with a SIB strategy from January 2022 onward, in accordance with a change in departmental protocol.

Exclusion criteria were histology other than squamous cell carcinoma, treatment with three-dimensional conformal radiotherapy (3D-CRT), metastatic or oligometastatic disease at diagnosis, omission of extended pelvic nodal irradiation including the inguinal regions, palliative treatment intent, inclusion in a clinical trial, and irradiation regimens outside the scope of the present study.

### 2.2 Pre-therapeutic imaging

All patients underwent baseline staging with contrast-enhanced pelvic magnetic resonance imaging (MRI) and 18F-fluorodeoxyglucose positron emission tomography (FDG-PET).

### 2.3 Radiotherapy protocols

All patients were treated on two linear accelerators (Clinac® and TrueBeam®, Varian Medical Systems) using image-guided volumetric modulated arc therapy (IGRT-VMAT) with 6 MV photon beams.

In both groups, the prescribed dose to the primary tumour and involved lymph nodes was 60 Gy in 30 fractions of 2 Gy, delivered once daily, 5 fractions per week. In the sequential group, elective nodal irradiation was delivered to 44 Gy in 22 fractions of 2 Gy. In the SIB group, the prophylactic nodal dose was 45 Gy in 30 fractions of 1.5 Gy.

The gross tumour volume (GTV) included the primary tumour (GTV-T) and involved lymph nodes (GTV-N). Target delineation followed the French RECORAD recommendations [15], [16]. For the primary tumour, the clinical target volume (CTV-T) included the involved anal canal and rectal wall over the cranio-caudal extent of the tumour, together with a 5 mm expansion of the GTV to account for microscopic extension. Elective nodal CTV encompassed the mesorectal, presacral, internal iliac, external iliac, obturator and inguinal nodal regions, as well as the bilateral ischiorectal fossae. Involved lymph nodes received an additional 5 mm margin to generate the nodal CTV (CTV—N).

The planning target volume (PTV) was generated by adding a 5 mm isotropic margin to the CTV. In the sequential boost group, the first treatment phase (PTV1) corresponded to the elective pelvic irradiation (44 Gy in 22 fractions), followed by a sequential boost phase (PTV2) delivering an additional 16 Gy in 8 fractions to the primary tumour and involved lymph nodes, for a total dose of 60 Gy. The interval between PTV1 and PTV2 was left to the treating physician's discretion according to the patient's clinical condition and acute treatment-related adverse events. No additional fractions or dose compensation were delivered in the event of treatment interruption. To better characterise OTT, the durations of PTV1, the interval between PTV1 and PTV2, and the duration of PTV2 were retrospectively collected from the treatment records.

The prescribed radiotherapy doses were identical regardless of disease stage. Patients with both localised and locally advanced disease received the same dose to the primary tumour and involved lymph nodes. Differences between treatment groups were restricted to the elective nodal irradiation technique (sequential boost or SIB).

Radiotherapy was delivered once daily, 5 fractions per week, from Monday to Friday, with no treatment during weekends, public holidays, or scheduled machine maintenance days. No additional fractions or dose compensation were delivered in case of treatment interruption.

### 2.4 Chemotherapy

Concurrent chemoradiotherapy was indicated for patients with nodal involvement (N+) or with T2 tumours >3 cm to T4 disease without nodal involvement (N0). Chemotherapy consisted of continuous infusion 5-fluorouracil (5-FU) at 1000 mg/m^2^/day over 96 h (days 1–4 and 29–32), combined with mitomycin C (MMC) administered as an intravenous bolus at 10 mg/m^2^ on days 1 and 29, for a total of two cycles. As an alternative to 5-FU, capecitabine (Xeloda®) was administered at a dose of 825 mg/m^2^ twice daily on radiotherapy days.

Dose reduction or treatment interruption was allowed based on the patient's clinical condition and treatment-related adverse events, particularly hematological adverse events. All patients were screened for dihydropyrimidine dehydrogenase (DPD) deficiency prior to initiation of fluoropyrimidine-based chemotherapy.

Induction chemotherapy was administered at the physician's discretion in selected patients with bulky T4 tumours or T3 N+ disease.

### 2.5 Clinical evaluation and follow-up

Acute and late treatment-related adverse events, including cutaneous, gastrointestinal, urinary adverse events and lymphopenia, were graded according to the Common Terminology Criteria for Adverse Events (CTCAE), version 5.0. The highest grade observed for each treatment-related adverse events category was recorded. Treatment-related adverse events occurring within 90 days after completion of radiotherapy were considered as acute, whereas those occurring thereafter were considered late.

The first post-treatment evaluation was performed four months after treatment completion and included clinical examination, pelvic magnetic resonance imaging (MRI), and biological assessment including a complete blood count, liver function tests, serum calcium measurement, and serum squamous cell carcinoma (SCC) antigen levels. Patients were subsequently followed every 4 months for two years, and then every 6 months for an additional 3 years. Thoraco-abdominopelvic computed tomography (CT) and/or pelvic MRI was performed at least annually. Follow-up was conducted alternately by a radiation oncologist and a proctologist.

### 2.6 Endpoints

The primary endpoint was DFS, defined as the time from the end of radiotherapy to the first documented disease recurrence, death from any cause, or last follow-up in the absence of an event.

Recurrences were classified as local (anal canal), regional (mesorectal and/or pelvic and inguinal nodal regions), or distant (metastatic disease).

Secondary endpoints included OS and the incidence of acute and late treatment-related adverse events. OS was defined as the time from the end of radiotherapy to death from any cause or last follow-up.

### 2.7 Statistical analysis

DFS and OS were estimated using the Kaplan-Meier method and compared between groups with the log-rank test. Categorical variables were compared using the chi-square test or Fisher's exact test, as appropriate. Continuous variables were described as median and interquartile range (IQR) and compared using the Mann-Whitney *U* test.

Univariate and multivariable analyses were performed using Cox proportional hazards regression models. Covariates evaluated in the univariable analysis included sex, age at diagnosis, T stage, N stage, performance status (ECOG/WHO), disease stage group [early-stage disease (T1-T2, N0) versus advanced disease (T3-T4 and/or N+)], p16 status, HIV status, smoking status, body mass index, use of induction chemotherapy, delivery of concurrent chemotherapy, number of mitomycin C cycles, delivery of a nodal boost, number of boost volumes, OTT, and lymphocyte count before treatment and at 1–3 months after completion of radiotherapy. Absolute lymphocyte counts were retrospectively collected from routine complete blood counts performed before treatment and at the first post-treatment evaluation, when available. As patients were managed on an outpatient basis, blood tests were performed as part of routine clinical care. Because laboratory results were not systematically incorporated into the hospital medical records, complete paired lymphocyte measurements were not available for all patients.

For DFS, variables associated with the outcome at *p* < 0.10 in univariable analysis were considered for inclusion in the multivariable model. For OS, given the limited number of events, the multivariable model was constructed using clinically relevant covariates selected a priori based on their prognostic importance and potential confounding effect. When both continuous and dichotomised forms of the same variable were available, the continuous variable was preferentially retained in the multivariable model to preserve statistical power and avoid collinearity. OTT was retained because it represented a clinically relevant potential confounder of the association between radiotherapy technique and oncological outcomes. Results are reported as hazard ratios (HRs) with 95% confidence intervals (CIs).

All tests were two-sided, and a *p*-value <0.05 was considered statistically significant. Statistical analyses were performed using Stata version 15.1 (StataCorp, College Station, TX, USA).

## 3 Results

### 3.1 Study population

**Fig. 1:**
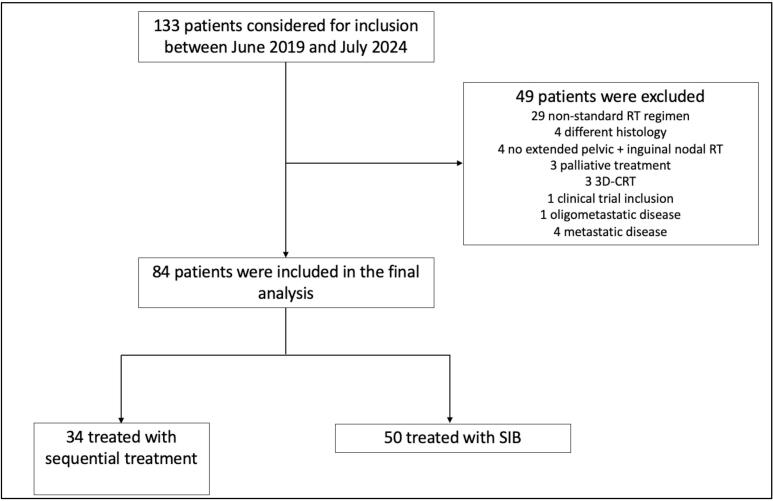
Study Flow Chart. Selection process of patients with anal squamous cell carcinoma treated with VMAT between 2019 and 2024. Abbreviations: RT = radiotherapy; 3D-CRT = three-dimensional conformal radiotherapy; SIB = simultaneous integrated boost.

Between June 2019 and July 2024, 133 patients were treated for anal canal cancer in the Department of Radiation Oncology at Saint-Louis Hospital, Paris. Of these, 49 were excluded from the analysis for the following reasons: non-standard radiotherapy regimen (*n* = 29), histology other than squamous cell carcinoma (*n* = 4), omission of extended pelvic nodal irradiation including the inguinal regions (n = 4), palliative treatment intent (*n* = 3), treatment with three-dimensional conformal radiotherapy (3D-CRT) (n = 3), inclusion in a clinical trial (*n* = 1), oligometastatic disease at diagnosis (n = 1), and metastatic disease at diagnosis (*n* = 4). A total of 84 patients were included in the final analysis, of whom 50 were treated with a SIB technique and 34 with a sequential radiotherapy approach (Fig. 1).

### 3.2 Patient, tumour, and treatment characteristics

Patient, tumour, and treatment characteristics are summarized in Table 1.

**Table 1:** Baseline patient, tumour and treatment characteristics.

	Sequential RT (*n* = 34)	SIB (*n* = 50)	*p*-value
Gender, n (%) Male Female	11 (32%) 23 (68%)	12 (24%) 38 (76%)	0.459
Age at Diagnosis (years; Median)	66.5 [52–69]	67.5 [56–76]	0.185
Age, n (%) < 55 years ≥ 55 years	10 (29%) 24 (71%)	10 (20%) 40 (80%)	0.434
Weight at Diagnosis (Kg)	66.5 [60–84]	65.0 [57–73]	0.255
BMI at Diagnosis (kg/m^2^)	24.2 [21.1–29.1]	23.0 [21.6–25.6]	0.158
T stage, n (%) Tx T1 T2 T3 T4	1 (3%) 2 (6%) 14 (41%) 13 (38%) 4 (12%)	0 (0%) 6 (12%) 22 (44%) 16 (32%) 6 (12%)	0.639
N stage, n (%) N0 N + Nx	11 (32%) 21 (62%) 2 (6%)	21 (42%) 29 (58%) 0 (0%)	0.175
PS, n (%) 0–1 ≥ 2 Missing	29 (85%) 3 (9%) 2 (6%)	45 (90%) 4 (8%) 1 (2%)	0.753
Prognosis categories Early (T1-T2, N0) Advanced (T3-T4 or N+) Missing	9 (26%) 22 (65%) 3 (9%)	16 (32%) 34 (68%) 0 (0%)	0.128
p16 expression Positive Negative Missing	10 (29%) 0 (0%) 24 (71%)	26 (52%) 1 (2%) 23 (46%)	0.054
HIV status, n (%) Positive Negative	8 (24%) 26 (76%)	11 (22%) 39 (78%)	1.0
Tobacco smoking history Yes No Missing	15 (44%) 17 (50%) 2 (6%)	25 (50%) 22(44%) 3 (6%)	0.875
Induction CT Yes No	4 (12%) 30 (88%)	5 (10%) 45 (90%)	1.0
Treatment duration (days; Median,)	50 [48–55]	48 [45–49]	0.003
Treatment duration (Days) < 50 ≥ 50	15 (44%) 19 (56%)	39 (78%) 11 (22%)	0.002
LN Boost Yes no	18 (53%) 16(47%)	19 (38%) 31(62%)	0.188
LN Boost < 3LN ≥ 3LN	25 (74%) 9 (26%)	42 (84%) 8 (16%)	0.277
Concurrent chemotherapy, n (%) Yes No	29 (85%) 5 (15%)	41 (82%) 9 (18%)	0.158
Mitomycin C cycles⁎, n (%) 1 cycle 2 cycles	3 (9%) 26 (76%)	6 (12%) 35 (70%)	0.803
Lymphocytes counts before treatment (G/L)	1.6 [1.2–2.0]	1.7 [1.3–2.2]	0.250
Lymph 1–3 months after treatment (G/L)	0.7 [0.6–0.9]	0.8 [0.7–1.1]	0.029

BMI = body mass index; T = tumour; N = node; PS = performance status; HIV = human immunodeficiency virus; CT = chemotherapy; LN = lymph node. *p*-values in bold indicate statistical significance (*p* < 0.05).

⁎ Among patients who received concurrent chemotherapy.

Baseline demographic and clinical characteristics were generally well balanced between the two groups. The study population was predominantly female, with a median age at diagnosis of 66.5 years in the sequential group and 67.5 years in the SIB group. Most patients had a good performance status (ECOG 0–1). Rates of HIV infection and smoking history were similar between groups.

Tumour characteristics, including T and N stage, were comparable across groups, with locally advanced disease accounting for approximately two-thirds of cases. The proportion of patients with nodal involvement did not differ significantly between groups. p16 positivity was more frequent in the SIB group, although the difference did not reach statistical significance.

Regarding treatment characteristics, induction chemotherapy was administered in a minority of patients, with similar proportions in the two groups (12% vs 10%). A nodal boost was more frequently delivered in the sequential group than in the SIB group (53% vs 38%), although this difference was not statistically significant. The median OTT was significantly shorter in the SIB group than in the sequential group (48 vs 50 days), and a greater proportion of patients in the SIB group completed radiotherapy within 50 days (78% vs 44%, *p* = 0.002). In the sequential group, the median duration of PTV1 was 35 days, followed by a median interval of 3 days before PTV2, which lasted a median of 12 days (Supplementary Table S1).

### 3.3 Primary endpoint

Median follow-up from the end of radiotherapy was 21 months (95% CI, 19–26) in the SIB group and 48 months (95% CI, 40–58) in the sequential group.

**Fig. 2:**
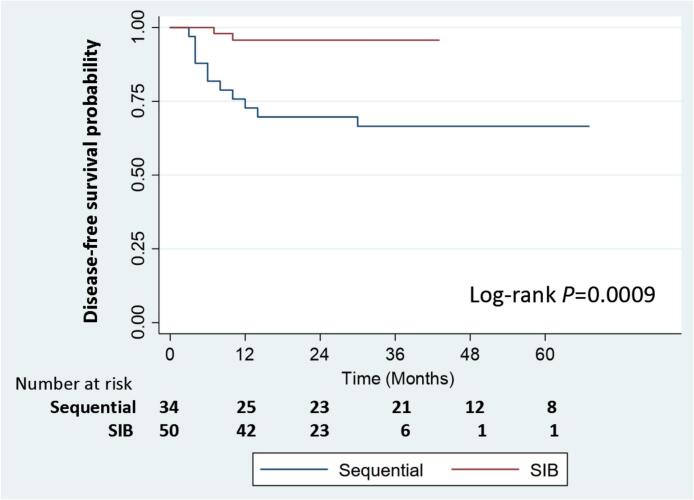
Kaplan-Meier estimates of disease-free survival according to radiotherapy technique. Numbers at risk are shown below the x-axis. Differences between groups were assessed using the log-rank test.

The 12-, 24-, and 36-month DFS rates were 96%, 96%, and 96% in the SIB group, compared with 73%, 70%, and 67% in the sequential group, respectively (log-rank *P* = 0.0009) (Fig. 2).

### 3.4 Univariable analysis

Thirteen recurrences were observed overall: 11/34 patients (32.4%) in the sequential group and 2/50 patients (4.0%) in the SIB group. In univariable Cox regression analysis, treatment with SIB was associated with a significantly lower risk of recurrence (HR 0.12, 95% CI 0.03–0.53; *p* = 0.006). Older age was also associated with improved DFS, both for age ≥ 55 years (HR 0.22, 95% CI 0.07–0.64; p = 0.006) and age ≥ 60 years (HR 0.30, 95% CI 0.10–0.88; *p* = 0.029). Longer OTT was associated with an increased risk of recurrence, both as a continuous variable (HR 1.10 per day, 95% CI 1.00–1.21; *p* = 0.046) and when treatment duration exceeded 50 days (HR 3.11, 95% CI 1.02–9.55; *p* = 0.047). The number of boost volumes was also associated with an increased risk of recurrence (HR 1.28, 95% CI 1.09–1.51; *p* = 0.002) (Table 4).

### 3.5 Multivariable analysis

In multivariable Cox regression analysis, treatment with SIB remained independently associated with improved DFS (HR 0.16, 95% CI 0.03–0.75; *p* = 0.020). Older age was also independently associated with improved DFS (HR 0.94 per year increase, 95% CI 0.90–0.98; *p* = 0.005), whereas OTT was not independently associated with recurrence (HR 1.04, 95% CI 0.92–1.18; *p* = 0.520). A higher number of boost volumes remained associated with an increased risk of recurrence (HR 1.20, 95% CI 1.02–1.40; *p* = 0.029) (Table 5).

### 3.6 Patterns of recurrence

Because only two recurrences occurred in the SIB group, meaningful statistical comparison of recurrence patterns between treatment groups was not possible.

### 3.7 Secondary endpoints

#### 3.7.1 Overall survival

**Fig. 3:**
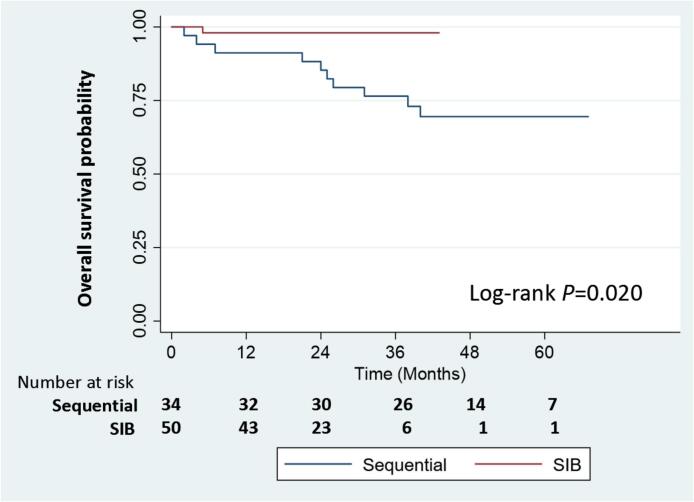
Kaplan-Meier estimates of overall survival according to radiotherapy technique. Numbers at risk are shown below the x-axis. Differences between groups were assessed using the log-rank test.

Overall survival (OS) rates at 12, 24, and 36 months were 91%, 85%, and 77%, respectively, in the sequential group, compared with 98%, 98%, and 98% in the SIB group. Kaplan-Meier analysis showed significantly improved OS in the SIB group compared with the sequential group (log-rank *p* = 0.020) (Fig. 3).

One death occurred in the SIB group (2.0%), compared with 10 deaths in the sequential group (29.4%).

In univariable Cox regression analysis, SIB was associated with improved OS (HR 0.12, 95% CI 0.01–0.98; *p* = 0.048), whereas older age, poorer performance status, induction chemotherapy, longer OTT, and a higher number of boost volumes were associated with worse OS (Table 6). In multivariable analysis, older age (HR 0.94 per year, 95% CI 0.90–0.98; *p* = 0.008), poorer performance status (HR 6.37, 95% CI 1.74–23.33; *p* = 0.005), and longer OTT (HR 1.21 per day, 95% CI 1.03–1.43; *p* = 0.022) remained independently associated with OS. Although SIB remained associated with a lower risk of death (HR 0.12), this association no longer reached statistical significance after adjustment (HR 0.12, 95% CI 0.01–1.31; *p* = 0.082) (Table 7).

In additional Cox regression analyses, neither concurrent chemotherapy nor the number of mitomycin C cycles was significantly associated with DFS or OS.

### 3.8 Treatment-related adverse events

Treatment-related adverse events analyses were based on available data.

Acute grade ≥ 2 skin treatment-related adverse events was observed in 29/33 patients (87.9%) in the sequential group and 36/50 patients (72.0%) in the SIB group, with a non-significant trend toward lower rates with SIB (*p* = 0.107). Acute grade ≥ 2 gastrointestinal treatment-related adverse events occurred in 20/34 patients (58.8%) in the sequential group and 22/50 patients (44.0%) in the SIB group (*p* = 0.266). Acute grade ≥ 2 urinary treatment-related adverse events was more frequent in the SIB group, occurring in 6/49 patients (12.2%) versus 2/34 patients (5.9%) in the sequential group, without reaching statistical significance (*p* = 0.462) (Table 2).

**Table 2:** Acute adverse event.

Acute adverse event (≥G2)	Sequential RT	SIB	p (Fisher)
Skin	29/33 (87.9%)	36/50 (72,0%)	0.107
Gastrointestinal	20/34 (58.8%)	22/50 (44,0%)	0.266
Urinary	2/34 (5.9%)	6/49 (12,2%)	0.462

Late grade ≥ 2 skin treatment-related adverse events occurred in 3/29 patients (10.3%) in the sequential group and 8/46 patients (17.4%) in the SIB group (*p* = 0.513). Late grade ≥ 2 gastrointestinal treatment-related adverse events was reported in 4/29 patients (13.8%) in the sequential group and 3/47 patients (6.4%) in the SIB group (*p* = 0.417). No late grade ≥ 2 urinary treatment-related adverse events was observed in either group (Table 3).

**Table 3:** Late adverse event.

Late adverse event (≥G2)	Sequential RT	SIB	p (Fisher)
Skin	3/29 (10.3%)	8/46 (17.4%)	0.513
Gastrointestinal	4/29 (13.8%)	3/47 (6.4%)	0.417
Urinary	0/29 (0.0%)	0/46 (0.0%)	1.000

**Table 4:** Univariable Cox proportional hazards regression analysis for disease-free survival.

Variable	Hazard ratio	95% CI	*p* value
Sex	0.76	0.23–2.46	0.643
Smoking	1.00	0.32–3.09	0.993
Age ≥ 55 years	0.22	0.07–0.64	0.006
Age ≥ 60 years	0.30	0.10–0.88	0.029
WHO performance status	1.54	0.67–3.54	0.307
WHO PS ≥2	1.24	0.16–9.59	0.839
Locally advanced disease	2.48	0.54–11.32	0.241
p16 positivity	Not estimable	–	1.000
HIV infection	1.11	0.31–4.04	0.872
Severe toxicity	0.34	0.06–1.84	0.208
BMI	1.03	0.92–1.16	0.575
Induction chemotherapy	2.54	0.56–11.59	0.229
Number of induction chemotherapy cycles	1.27	0.87–1.83	0.211
Overall treatment time (continuous)	1.10	1.00–1.21	0.046
Boost GTV/GTV-N	2.31	0.76–7.07	0.135
Number of boost volumes	1.28	1.09–1.51	0.002
SIB	0.12	0.03–0.53	0.006
Concurrent chemotherapy	1.00	0.22–4.53	0.998
Number of MMC cycles	0.90	0.45–1.81	0.776

**Abbreviations:** BMI, body mass index; CI, confidence interval; DFS, disease-free survival; HR, hazard ratio; MMC, mitomycin C; SIB, simultaneous integrated boost; WHO PS, World Health Organization performance status; GTV, Gross Tumour Volume; N, Nodes.

**Table 5:** Multivariable Cox proportional hazards regression analysis for disease-free survival.

Variable	Hazard Ratio (HR)	95% CI	*p* value
Age (per year increase)	0.94	0.90–0.98	0.005
Overall treatment time (per day increase)	1.04	0.92–1.18	0.520
Number of boost volumes	1.20	1.02–1.40	0.029
Simultaneous integrated boost (SIB)	0.16	0.03–0.75	0.020

**Abbreviations:** CI, confidence interval; DFS, disease-free survival; HR, hazard ratio; SIB, simultaneous integrated boost.

Variables included in the multivariable model were selected from univariable analyses (*p* < 0.10). Overall treatment time was retained as a clinically relevant potential confounder of the association between radiotherapy technique and disease-free survival.

**Table 6:** Univariable Cox proportional hazards regression analysis for overall survival.

Variable	Hazard ratio (HR)	95% CI	*p* value
Female sex	0.26	0.08–0.85	0.027
Age (per year increase)	0.94	0.91–0.99	0.010
Nodal involvement (N+)	7.11	0.91–55.62	0.062
WHO/ECOG performance status	3.02	1.37–6.64	0.006
Locally advanced disease	4.67	0.59–36.94	0.144
p16 status	–	–	1.000⁎
HIV infection	3.25	0.99–10.67	0.051
Smoking	1.05	0.30–3.66	0.933
Body mass index (BMI)	1.01	0.90–1.14	0.896
Induction chemotherapy	6.21	1.58–24.36	0.009
Overall treatment time (per day increase)	1.18	1.06–1.31	0.003
Delivery of nodal boost	2.38	0.70–8.14	0.156
Number of boost volumes	1.37	1.18–1.59	<0.001
Simultaneous integrated boost (SIB)	0.12	0.01–0.98	0.048
Concurrent chemotherapy	0.75	0.16–3.49	0.716
Number of mitomycin C cycles	0.86	0.40–1.83	0.694
			

⁎ The p16 analysis was not informative because of quasi-complete separation (very limited number of events).

**Table 7:** Multivariable Cox proportional hazards regression analysis for overall survival.

Variable	HR	95% CI	*p*
Age (continuous)	0.94	0.90–0.98	0.008
ECOG/WHO performance status	6.37	1.74–23.33	0.005
Overall treatment time (continuous)	1.21	1.03–1.43	0.022
SIB	0.12	0.01–1.31	0.082

**Abbreviations:** CI, confidence interval; ECOG, Eastern Cooperative Oncology Group; HR, hazard ratio; OS, overall survival; WHO PS, World Health Organization performance status; SIB, simultaneous integrated boost.

Variables included in the multivariable model were selected based on their clinical relevance and results of the univariable analyses. Overall treatment time was retained as a clinically relevant potential confounder of the association between radiotherapy technique and overall survival.

No acute or late grade > 3 skin, gastrointestinal, or urinary treatment-related adverse events was observed in either group.

Post-treatment lymphocyte counts were significantly higher in the SIB group than in the sequential group (median 0.82 vs 0.68 G/L; *p* = 0.029), whereas baseline lymphocyte counts and treatment-related lymphocyte variation did not differ significantly between groups. However, these data should be interpreted with caution because of missing values.

## 4 Discussion

In this single-centre retrospective study, the use of a SIB was associated with a significant improvement in DFS compared with a sequential radiotherapy approach. This association remained significant in multivariable analysis, suggesting a potential independent effect of treatment delivery strategy on tumour control.

Importantly, this benefit was observed despite similar tumour dose prescriptions between groups and without a meaningful increase in nodal biological dose, indicating that dose escalation alone does not explain the observed differences. The SIB technique was associated with a shorter OTT (median 48 vs 50 days, *p* = 0.003), and a greater proportion of patients completed radiotherapy within 50 days (78% vs 44%, *p* = 0.002), reflecting the more integrated delivery of elective and boost irradiation. Although prolonged OTT has been associated with accelerated clonogenic repopulation and poorer local tumour control [17], our multivariable analysis showed that OTT was not independently associated with DFS after adjustment, whereas the association between SIB and improved DFS remained statistically significant. These findings suggest that the benefit associated with SIB cannot be explained solely by a modest reduction in treatment duration and that other treatment-related factors or residual confounding may also have contributed to the observed differences.

Previous studies have consistently demonstrated the detrimental impact of prolonged OTT in anal canal cancer. In the pooled PARADAC analysis, longer treatment duration was associated with increased locoregional recurrence and reduced survival outcomes, independently of total dose [18]. Similarly, ACT II demonstrated that treatment duration exceeding 42 days was associated with decreased progression-free and OS, consistent with pooled analyses of RTOG 87–04 and RTOG 98–11 and with the study by Mehta et al. [19], [20], [21]. In our study, the SIB group had a significantly shorter OTT; however, the absolute difference between groups was modest (median 48 vs. 50 days).

Although short treatment interruptions have not always been associated with poorer outcomes [22], [23], [24], prolonged or repeated interruptions and treatment non-completion have consistently been linked to worse outcomes and a higher need for salvage surgery.

Therefore, the shorter treatment duration observed with SIB may have contributed, at least in part, to improved tumour control.

Treatment interruptions during chemoradiotherapy are mainly driven by acute chemoradiotherapy-related adverse events, particularly cutaneous, gastrointestinal, and hematological events, as well as by patient- and organization-related factors [25], [26], [27]. Advances in IMRT have reduced severe acute treatment-related adverse events and treatment interruptions [28], [29]. In our cohort, SIB was associated with a trend toward lower acute treatment-related adverse events without an increased clinically relevant late adverse events, which may have contributed to a more continuous treatment delivery.

Nevertheless, the persistence of the SIB effect after adjustment for OTT suggests that additional factors related to treatment delivery, dosimetry, or unmeasured confounders may also have contributed to the observed improvement in DFS.

The role of SIB should also be interpreted in the context of evolving dose de-escalation strategies. In the phase II RTOG-0529 trial [8], SIB-IMRT with reduced elective nodal doses achieved favorable long-term oncological outcomes, with no isolated nodal recurrences, findings supported by other series [30], [31], [32], [33]. Historical trials and recent IMRT series using conventional fractionation have reported comparable outcomes [34], [35], and direct comparisons remain limited, with retrospective data suggesting no major survival differences between SIB and sequential approaches [36]. RTOG-0529 also reported reduced acute treatment-related adverse events with SIB-IMRT compared with historical controls, although its primary endpoint was not met [8], [29].

More recently, risk-adapted de-escalation strategies have shown promising results. The randomised PLATO-ACT4 trial demonstrated that reduced-dose radiotherapy in early-stage disease achieved comparable complete response rates with improved tolerance and fewer treatment interruptions [14]. The ongoing ECOG-ACRIN DECREASE randomised phase II trial (NCT04166318) is evaluating further treatment de-intensification in patients with T1-T2 N0 anal canal cancer, with co-primary endpoints of maintaining 2-year disease control while improving patient-reported bowel function.

Regarding OS, although a significant benefit was observed in unadjusted analysis in favor of SIB, this association did not remain statistically significant after adjustment, likely due to the limited number of deaths, particularly in the SIB group. Performance status (ECOG ≥2) emerged as the main prognostic factor for mortality. The discrepancy between DFS and OS is not unexpected, as OS is influenced by competing risks and the effectiveness of salvage treatments.

Exploratory interaction analyses provided no evidence that the effect of the SIB technique differed according to disease stage (localised versus locally advanced); however, these findings should be interpreted with caution given the limited number of recurrence events and the resulting low statistical power.

The higher post-treatment lymphocyte counts observed in the SIB group suggest a potential immunological effect, although the clinical significance of this finding remains uncertain. In our exploratory analyses, neither baseline nor post-treatment absolute lymphocyte counts were significantly associated with disease recurrence. However, these analyses were limited by the small number of events and incomplete paired lymphocyte data. This finding should be interpreted with caution. Therefore, although SIB appeared to better preserve circulating lymphocyte counts, our data do not support an independent association between lymphocyte counts and disease recurrence. Although data in anal canal cancer remain limited and heterogeneous, several studies have suggested a prognostic impact of treatment-related lymphopenia. Severe lymphopenia has been associated with poorer outcomes in some series [37] [38], although this relationship is not consistently observed [39]. More broadly, meta-analyses have suggested an overall detrimental impact of radiation-induced lymphopenia across several solid tumours, with variability according to tumour type and study design [40], [41].

From a radiobiological perspective, lymphocytes are highly radiosensitive, and treatment-induced lymphopenia likely depends not only on bone marrow irradiation but also on dose delivered to circulating blood and immune-related structures [42]. In pelvic malignancies, bone marrow exposure may also play an important role [43]. Modern IMRT techniques, including SIB, may therefore help reduce the integral dose to normal tissues and limit irradiation of lymphoid tissues.

Nevertheless, direct evidence comparing SIB and sequential boost strategies in terms of lymphopenia remains lacking, and any potential immunological advantage of SIB therefore remains hypothetical. Prospective studies incorporating longitudinal immune monitoring together with dedicated bone marrow and immune-organ dosimetry are warranted to better clarify these relationships.

## 5 Strengths and limitations

This study benefits from a relatively homogeneous cohort treated within a single-centre using standardised protocols and modern VMAT techniques. The single-centre design, while limiting generalizability, ensured high consistency in volume contouring and dose-painting strategies, reducing inter-observer variability. Furthermore, the use of multivariable adjustment helped account for potential imbalances in baseline characteristics.

However, several limitations must be acknowledged. Its retrospective nature may introduce selection and information bias, and the absence of randomisation limits formal causal inference. The limited sample size and number of events reduce statistical power, particularly for OS analysis. Additionally, the shorter follow-up in the SIB group is a notable limitation; however, since the risk of recurrence in anal canal cancer is highest within the first 24 to 36 months, a period that was adequately covered by our median follow-up, the observed benefit in DFS remains clinically relevant. Although the implementation of the SIB protocol coincided with the later treatment period, baseline patient and tumour characteristics remained comparable between treatment periods, indicating that no major changes in patient selection or overall clinical management occurred over time. Importantly, radiotherapy practice otherwise remained stable throughout the study period, including VMAT delivery with 6-MV photons, target delineation policy, prescribed tumour dose, image guidance, concurrent chemotherapy, and treatment on the same linear accelerators according to machine availability. Nevertheless, because treatment period and the implementation of the SIB protocol were intrinsically linked, residual temporal confounding cannot be completely excluded. Furthermore, although SIB remained independently associated with improved DFS after multivariable adjustment, the magnitude of the observed effect should be interpreted cautiously, as the modest difference in OTT between groups suggests that additional unmeasured confounding factors may also have contributed to the observed association. Similarly, the imbalance in p16 positivity between treatment groups, together with the relatively large proportion of missing p16 data, represents another potential source of residual confounding.

In addition, the analysis of lymphocyte counts should be interpreted with caution. Owing to the retrospective real-world design, complete paired lymphocyte measurements were unavailable for all patients because the results of routine blood tests were not systematically available in patients' hospital records. Furthermore, post-treatment lymphocyte counts were obtained at the first routine post-treatment evaluation and therefore were not collected according to a strictly predefined study schedule. Finally, the absence of dedicated dosimetric analyses of bone marrow and other immune-related structures, together with missing longitudinal lymphocyte data, further limits the interpretation of the exploratory immune-related findings.

## 6 Conclusion

In this single-centre retrospective cohort, a SIB was associated with significantly improved DFS in localised or locally advanced anal canal cancer, without an increase in treatment-related adverse events. The shorter OTT and potential immune-sparing effects enabled by this approach likely contributed to the observed oncological benefit. These results support the use of SIB as a robust delivery strategy, consistent with its adoption as the technical standard in modern trial designs such as the PLATO program. While PLATO focuses on dose personalization, our findings highlight that the delivery method itself, by optimising the therapeutic ratio, remains a critical factor. Larger prospective multicentre studies are warranted to further refine the impact of treatment duration and dose distribution on long-term survival and quality of life.

## CRediT authorship contribution statement

**Cécile Giannetta:** Writing – review & editing, Writing – original draft, Formal analysis, Data curation. **Rafik Nebbache:** Writing – review & editing. **Isabelle Etienney:** Writing – review & editing, Resources. **Gael Goujon:** Writing – review & editing, Resources. **Thomas Aparicio:** Writing – review & editing, Resources. **Léon Maggiori:** Writing – review & editing, Resources. **Laurent Abramowitz:** Writing – review & editing, Writing – original draft, Validation, Supervision, Resources, Methodology, Formal analysis, Conceptualization.

## Declaration of competing interest

The authors declare that they have no known competing financial interests or personal relationships that could have appeared to influence the work reported in this paper.
