# Supplementary material for: Simultaneous integrated boost vs sequential boost chemoradiotherapy in anal squamous cell carcinoma: Association with improved disease-free survival and the role of treatment delivery parameters
Source: Clin Transl Radiat Oncol. 2026 Jul 7;60:101234. doi: 10.1016/j.ctro.2026.101234 (PMC13380012; doi:10.1016/j.ctro.2026.101234)
Supplement: Supplementary file 1 — Supplementary material [file mmc1.docx]

**Supplementary Table S1**. Duration of PTV1, interval between PTV1 and PTV2, and PTV2 in the sequential boost group

| **Sequential boost** | **Median (IQR)** |
| --- | --- |
| PTV1 duration (days) | 35 (34–36) |
| Interval PTV1–PTV2 (days) | 3 (2–5) |
| PTV2 duration (days) | 12 (11–13) |
| Overall treatment time (days) | 50 (48–53) |

**Abbreviations:** IQR, interquartile range; OTT, overall treatment time; PTV, planning target volume.
